# Characterization of Nestin, a Selective Marker for Bone Marrow Derived Mesenchymal Stem Cells

**DOI:** 10.1155/2015/762098

**Published:** 2015-07-06

**Authors:** Liang Xie, Xin Zeng, Jing Hu, Qianming Chen

**Affiliations:** State Key Laboratory of Oral Diseases, West China School of Stomatology, Sichuan University, Chengdu, Sichuan 610041, China

## Abstract

Mesenchymal stem cells (MSCs) are multipotent cells capable of differentiating into multiple cell lineages and contributing to tissue repair and regeneration. Characterization of the physiological function of MSCs has been largely hampered by lack of unique markers. Nestin, originally found in neuroepithelial stem cells, is an intermediate filament protein expressed in the early stages of development. Increasing studies have shown a particular association between Nestin and MSCs. Nestin could characterize a subset of bone marrow perivascular MSCs which contributed to bone development and closely contacted with hematopoietic stem cells (HSCs). Nestin expressing (Nes^+^) MSCs also play a role in the progression of various diseases. However, Nes^+^ cells were reported to participate in angiogenesis as MSCs or endothelial progenitor cells (EPCs) in several tissues and be a heterogeneous population comprising mesenchymal cells and endothelial cells in the developing bone marrow. In this review article, we will summarize the progress of the research on Nestin, particularly the function of Nes^+^ cells in bone marrow, and discuss the feasibility of using Nestin as a specific marker for MSCs.

## 1. Introduction

Adult stem cells as early stage and undifferentiated cells are capable of developing into various types of cells and preserve the potential for regenerating or repairing damaged tissues [1]. More and more stem cells in different tissues have been recognized including neural stem cells (NSCs) [2], HSCs [3], dental pulp stem cells (DPSCs) [4, 5], and other tissue stem cells. During embryonic development, three germ layers (mesoderm, endoderm, and ectoderm) exist and make up the entire body through differentiating into different lineages [6]. Mesoderm as cells of the middle layer in the embryo will develop into bone, muscle, blood, kidneys, connective tissue, and other related structures [7]. MSCs and HSCs are thought to be derived from mesoderm [8]. MSCs are an example of multipotent stem cells defined as nonhematopoietic, plastic-adherent, colony-forming cells and have the capacity to self-renew and differentiate into osteoblastic, chondrocytic, and adipocytic cells [9–11]. In 1991, the presence of MSCs in bone marrow was discovered by Caplan [12]. Thereafter, MSCs were successively isolated from many other tissues and organs, such as heart [13], lung [14], umbilical cord tissue [15], peripheral blood [16], adipose tissue [17], and muscle [18]. The umbilical cord tissue contains the youngest, most primitive MSCs that have a great value for clinical application [19]. Although many markers have been reported to identify MSCs, no single marker is unique and generally accepted (Table 1). Thus, their location, origination, and physiological functions* in vivo* have not been fully characterized. Nestin is an intermediate filament protein originally described as a NSCs marker that appeared during development of the central nervous system (CNS) and has been downregulated once Nes^+^ cells differentiate into neurons or glial cells [20–22]. Lendahl et al. first discovered this gene specifically expressed in neuroepithelial stem cells distinguishing from the differentiated cells in the neural tube. Then Nestin was found to be expressed not only in NSCs, but also in many other types of cells including endothelial cells [23], cancer cells [24], fibroblasts [25], and other tissues such as tooth bud, testes, hair follicle sheath, skin, pancreas, and newly formed blood vessels [26–29]. Several studies have shown that cells that expressed the Nestin-GFP (Nes-GFP) transgene behave functionally as MSCs and are closely associated with HSC quiescence and maintenance in bone marrow [30–32]. A recent study, however, reported that Nes^+^ cells could mark both endothelial and nonendothelial cells during endochondral ossification [33]. Some research groups also claimed that Nestin was expressed in EPCs of different tissues [34]. Considering this complicated situation, in this review, we will analyze recent research evidence regarding Nestin as a marker of MSCs and discuss the specific function of Nes^+^ cells in bone marrow.

## 2. Basics of Nestin

Nestin is defined as a class VI intermediate filament protein [20]. Intermediate filaments are major components in cytoskeleton same as microtubules and microfilaments [35]. Based on their molecular structure, these proteins can be grouped into six main types (I–VI). Types I and II are acidic and basic keratin which can be subdivided into two groups: epithelial keratins and trichocytic keratins. Type III includes desmin, peripherin, vimentin, and glial fibrillary acidic protein (GFAP), which can form homo- or heteropolymeric proteins. Type IV contains four components: *α*-internexin, neurofilaments, synemin, and syncoilin. Type V is mainly nuclear lamins [36–38]. Nestin is the only protein in mammals comprised of type VI intermediate filaments with 1618 amino acids and a molecular weight of 176 kDa [39]. A short N-terminus and an unusually long C-terminus in Nestin structure result in complex binding to an array of structural proteins. N-terminus is known to be integrant for filament protein assembly [40]. The short N-terminus of Nestin leads to preferential intermediate filaments formation with heterodimers and heterotetramers rather than homodimers. The long C-terminal portion may function as links or bridges between intermediate filaments and microtubules [41, 42]. The expression of Nestin was reported to be upregulated in most mitotic cells and downregulated in all cells upon differentiation. The reason may be that Nestin is reorganized during mitosis by cdc2-mediated Thr^316^ phosphorylation [43].

## 3. Nestin in MSCs

Mesenchymal stem cells (MSCs) are multipotent cells capable of differentiating into multiple cell lineages such as osteoblasts, adipocytes, chondrocytes, and tenocytes [44]. The precise standardized protocols for isolation and expansion of these cells are still missing due to the lack of accepted unique marker. Sca1, Stro-1, CD73, CD90, CD105, and many other markers claimed specific for MSCs before are eventually proven to be unspecific [45]. Tondreau's group initially reported that BM-derived MSCs expressed Nestin before differentiation* in vitro* [46]. Wiese et al. showed that Nestin was enriched in ES-derived progenitor cells that could develop into neuroectodermal, endodermal, and mesodermal lineages [28]. Thereafter, Méndez-Ferrer et al. presented abundant evidence to confirm that bone marrow MSCs could be identified by Nestin [31]. They found Nes^+^ MSCs in the bone marrow contained all of the mesenchymal progenitor activity (fibroblastic colony-forming units, CFU-Fs) and had the potential of self-renewal and trilineage differentiation. Nes^+^ MSCs colocalized with HSCs supporting HSC maintenance and homing [47, 48]. Because of its intracellular expression in cells, the isolation of Nes^+^ MSCs for culture or even clinical therapy is still a challenge. Therefore, Pinho et al. suggested to combine cell surface proteins platelet-derived growth factor receptor-*α* (PDGFR-*α*), CD51 (also called ITGAV) for identifying Nes^+^ MSCs [49]. They validated that PDGFR-*α*, CD51, and Nestin were coexpressed on CD146^+^ cells which represented a subtype of MSCs in humans [50] and on a large cluster of perivascular stromal cells in mice. These cells in the bone marrow represented most MSCs that were essential for HSCs expansion. Notably, researchers found distinct Nes^+^ MSCs distributed on distinct vessels [30]. Based on cell fluorescence intensity and cellular morphology, two distinct types of Nes-GFP^+^ cells (Nes-GFP^bright^ and Nes-GFP^dim^) were identified and characterized. Both of them showed the mesenchymal progenitor activities. Although the number of Nes-GFP^bright^ cells was much lower than Nes-GFP^dim^ cells in bone marrow, Nes-GFP^bright^ cells contained most CFU-Fs and higher expression of genes which associated with the HSC niche. By contrast, DNA replication and cell cycle pathways were significantly enriched in Nes-GFP^dim^ cells. Another fundamental difference between these two subtypes was their physiological location that Nes-GFP^bright^ cells attached along arterioles, while Nes-GFP^dim^ cells were associated with sinusoids.

## 4. Nestin in HSCs Maintenance

An important role of bone marrow Nes^+^ MSCs was regulating HSCs traffic under homeostasis. These rare stromal cells helped to support the “niche” which formed by MSCs and HSCs in the bone marrow. Nes^+^ MSCs were innervated sympathetic nerve fibers and highly enriched in the key molecules of HSC maintenance, such as cytokines chemokine ligand 12 (CXCL12), stem cell factor (SCF), and Kit ligand (Kitl) [79]. NG2+ Nes-GFP^bright^ pericytes which were quiescent and chemoresistant ensheathed the arterioles for promoting HSC maintenance and retention within the bone marrow. In addition, Nes-GFP^dim^ cells which largely overlap with perivascular LepR^+^ stromal cells contribute to adipocyte and bone regeneration but are easily destroyed after chemotherapy [30, 32].

Conditional knockout or deletable animal models were widely used to eliminate key factors from niche cells for investigating the regulators of HSCs maintenance [80]. Méndez-Ferrer et al. performed selective ablation of Nes^+^ cells by using Cre-recombinase-inducible diphtheria toxin receptor lines and found that the number of bone marrow CD150+ CD48− LSK cells decreased rapidly. Moreover, the homing of hematopoietic progenitor to bone marrow markedly reduced [31]. Nes-GFP^+^ cells were shown to express high levels of SCF; however, deletion of SCF in another Nestin transgenic line (Nes-Cre mice), in which Cre recombinase was also driven by both promoter and intronic enhancer of Nestin gene, had no effect on HSC function [49, 80, 81]. The authors speculated that the reduction of SCF might be compensated by the nontargeted Nes^+^ and lineage cells because the recombination efficiencies among Nes-Cre^+^ cells in this model were quite low.

Altogether, these results clearly demonstrated that nestin^+^ MSCs as niche cells were essential for maintaining and mobilizing HSCs.

## 5. Nestin in Osteogenesis

In mammals, skeletal MSCs were suggested to differentiate into osteolineage cells and then participated in bone formation and HSCs maintenance. Nes-GFP cells and Nes-Cre-ER^T2^ linage cells exhibited mesenchymal stem/progenitor activities and contributed to osteogenesis in entire life. However, these cells showed different potential in different developmental stage. GFP-expressing osteoblasts and osteocytes were not detectable after a 1-month chase in adult Nes-Cre-ER^T2^ lines due to a slow turnover of the skeleton. They only can be observed after 8 months of lineage tracing [31]. Ono et al. tracked cell fates by using several triple-transgenic mice and showed that Nes^+^ cells in embryonic and postnatal bones were heterogeneous populations [33, 82]. Nestin expression in osteoblasts, endothelial lineages, and pericytes was detectable in the developing bone marrow. Nes-GFP^+^ MSCs had an active role in endochondral ossification. Another study conducted by Isern's group confirmed Nes^+^ cells preserved MSC activity throughout life and refined a quiescent subset of Nes^+^ cells derived from neural crest that preferably conserved MSC activity for helping to establish the HSC niche rather than contributing to endochondrogenesis [83].

These findings indicated that Nes^+^ MSCs possibly are more relevant to the endochondral ossification during development rather than the skeletal remodeling process in adult, which means Nes^+^ cells perform specific function in different stages of life.

## 6. Nestin in Angiogenesis

Due to the specific location of MSCs, researchers proposed a close association between MSCs and blood vessels. There is a putative concept that MSCs are likely to be an important inducer of angiogenesis by releasing angiogenic growth factors rather than directly differentiating into endothelial lineages, although some groups have successfully stimulated MSCs into endothelial cells* in vitro* [84–87]. Endothelial cells were commonly thought to be derived from EPCs that are distinct but contactable with MSCs [88]. Nestin has been reported to be expressed on both perivascular and endothelial cells during angiogenesis in several tissues by far. On the one hand, Klein et al. identified that Nes^+^ MSCs supported the tumor vessel maturation by differentiating into pericytes and smooth muscle cells through tumor-cell-secreted factors. These cells could be recruited to stabilize the tumor blood vessels angiogenesis by blocking vascular endothelial growth factor (VEGF) [89]. On the other hand, Nestin was considered as a novel early EPCs marker. Growing lines of evidence presented in different tissues such as brain [90], pancreas [91], and heart [92] have shown that Nestin is expressed in vascular EPCs and further closely associated with neovascularization. Early in 2002, Sugawara et al. detected a high expression of Nestin in proliferating endothelial cells* in vitro* [93]. They loaded physiologic levels of shear stress to endothelial cells and found a dramatic decrease in Nestin expression suggesting that Nestin could be a specific marker for proliferating endothelial cells in gliomas. Nestin was also reported to be expressed in tumor development including neurocytomas [94], pancreatic tumors [91], osteosarcomas [95], and gastrointestinal tumors [96, 97] and normally used as an angiogenesis marker in these tumors. However, based on the possibility that Nestin is expressed on both MSCs and EPCs, researchers should cautiously identify the real role of Nestin in order to instruct the translational and therapeutic study.

In the bone marrow, MSCs were shown to reside around blood vessels and capably differentiate into perivascular cells including pericytes and smooth muscle cells for stabilizing the neovasculature [98, 99]. Suzuki et al. assessed Nestin expression in bone marrow derived EPCs* in vitro* and concluded that Nestin was only expressed in proliferating EPCs, not in mature endothelial cells due to the downregulation at the transition of maturation [23]. Although the two studies mentioned above showed Nestin was expressed on endothelial cells in developing bone, more* in vivo* studies are needed to investigate the contribution of Nes^+^ cells as EPCs to angiogenesis during bone development and remodeling. Recently, Petrini et al. hypothesized the presence of mesodermal progenitor cells (MPCs) which are Nes^+^ CD31^+^ cell population in bone marrow. It is an interesting and reasonable hypothesis but still needs more evidence for the existence of MPCs and deeper investigation of their origin and physiological function [100–102].

Taken together, these studies suggested Nes^+^ cells may participate in angiogenesis as a heterogeneous cell population comprising MSCs and EPCs. Nes^+^ MSCs will form perivascular cells with Nes^+^ EPCs contributing to endothelial cells thereby and subsequently building blood vessel walls together.

## 7. Nestin in Diseases

Ever since Nestin was discovered, it seems that Nes^+^ cells play an important role in a lot of pathological changes or tissue repairing during disease progress [103]. Numerous studies have indicated that Nes^+^ bone marrow MSCs participate in many bone diseases' development, including osteoarthritis and acute myelogenous leukemia (AML) [104, 105]. Zhen et al. found large quantities of active TGF-*β*1 were released to the marrow during subchondral bone resorption and recruited Nes^+^ MSCs to form marrow osteoid islets and induced angiogenesis [104]. Knockout of TGF-*β* type II receptor (T*β*RII) in Nes^+^ MSCs reduced osteoarthritis development after ACLT surgery compared with sham-operated mice, which confirmed that Nes^+^ MSCs were the target cell population of TGF-*β* signaling. In the meantime, Hanoun's group showed that AML cells remodeled the bone marrow niche through abnormally inducing osteoblastic differentiation of bone marrow Nes-GFP^+^ cells with a block of osteoblastic maturation and consequently affecting healthy HSCs [105]. The study by Arranz et al. first analyzed Nestin expression in bone marrow samples from myeloproliferative neoplasms (MPNs) patients and discovered that Nestin^+^ MSCs might play a role in MPN. They engineered an associated mutation in Janus kinase 2 (JAK2) in HSCs and detected damage of the bone marrow niche through Nestin^+^ MSCs reduction, which further drive HSCs into neoplasia. Deletion of Nes^+^ MSCs caused a dramatic expansion of HSCs due to a reduction of CXCL12. It resulted in an indication of MPNs with an increase of hematopoietic progenitors in bone marrow, peripheral blood, and spleen, which was reversed by treatment with a *β*-adrenergic agonist [106, 107].

Coincidentally, Nes^+^ MSCs are not only involved in bone diseases progress, but also committed to other tissues injuries. Gao et al. investigated the role of MSCs in airway inflammation and lung regeneration and found that the increase of Nes^+^ MSCs in airway might be the main cause of asthma [108]. High levels of TGF*β*1 released from allergen-activated epithelium caused an excessive recruitment of Nes^+^ MSCs to the lungs. Furthermore, mobilization of Nes^+^ MSCs also participates in tissue repair and remodeling. Wan et al. showed that injured vessels would release growth factors to induce the migration of Nes^+^ MSCs which thereby contributed to vessel repair [109]. Nes^+^ MSCs were recruited to the injured parts from peripheral blood and differentiated into either endothelial cells or myofibroblastic cells for subsequent repairing. However, whether these Nes^+^ MSCs are mobilized from bone marrow needs further investigation.

Taken together, these studies suggested that Nestin could mark a subset of bone marrow MSCs that contribute to HSCs maintenance and tissue growth and regeneration.

## 8. The Controversy of Nestin

In contrast to the findings above, other scientists working on bone marrow MSCs raised doubts about whether Nestin could be a reliable marker for MSCs. Wong's group detected the expression of Nestin in equine, canine, and human bone marrow derived MSCs, respectively, during osteogenesis* in vitro*. They drew a conclusion that Nestin expression was maintained during osteogenic differentiation and, therefore, it is not a selective marker for MSCs [110]. By using Wnt1-Cre transgenic model and clonal analyses, Wislet-Gendebien et al. observed that Nes^+^ cells in the bone marrow were a mixed population containing neural crest stem cells (NCSCs) and MSCs [111]. MSCs could differentiate into functional neural cells under appropriate culture conditions by activating Wnt signaling pathway. A recent study by Zhou's group proposed that LepR could be a more specific marker for bone marrow MSCs because 94% of adult bone marrow CFU-Fs were formed by LepR^+^ cells [112]. They emerged and overlapped with SCF-GFP^+^, CXCL12-DsRed^high^, and Nes-GFP^dim^ cell population at the postnatal stage and then participated in most osteogenesis and adipogenesis in the adult bone marrow. The CFU-Fs and lineage tracing data showed Nes-Cre-ER expressing cells were not a significant source of bone* in vivo* although Méndez-Ferrer's group suggested previously that Nestin-CreER-expressing cells in the adult bone marrow could differentiate into osteoblasts and chondrocytes [31]. Furthermore, they found PDGFRa cells were derived from adult bone marrow cells that could be marked by LepR. LepR^+^ cells included Sca-1^−^ and Sca-1^+^ cells. PDGFRa^+^Sca-1^+^ cells, which are PaS cells, were highly efficient CFU-F-forming cells [49]. Conversely, PDGFRa^+^Sca-1^−^ cells were a subgroup of the CAR cells. There was only a small portion of the PaS, CAR, and LepR^+^ cells that overlapped with the Nes-GFP^dim^ cells. Gremlin 1 could be identified as a type of stem cells with bone, cartilage, and reticular stromal potential. Grem1^+^ cells and LepR^+^ cells were shown to be distinct from Nes-GFP^bright^ cells, but they expressed low levels of Nestin [113].

Apart from the relative concept for the expression level of Nes-GFP leading to these discrepancies, different constructs of Nestin transgenic models resulted in different cell types labeling. Nes-GFP transgenic mice were widely used in multiple tissue studies. The initial aim for establishing this model was to investigate the role of neural stem cells [114]. Mignone et al. combined the 5.8-kb promoter and the 1.8-kb second intronic enhancer of the Nestin gene to launch the transcription of the GFP protein for increasing the Nestin gene expression in the brain, in spite of several reports suggesting that expression of Nestin in the neuroepithelial cells of the developing embryo is dependent on the presence of a transcriptional enhancer that resides in the second intron of the gene. This resulted in brighter GFP signal for both Nes^+^ endothelial cells and Nes^+^ perivascular cells under the microscope [83]. Nes-Cre-ER^T2^ mouse model generated by Fishell's group was used by several groups for lineage tracing studies [115]. However, Nes-GFP and Nes-Cre-ER^T2^ lines had been reported to target different cell populations. Nes-GFP^+^ cells included both endothelial and mesenchymal cell types during bone development whereas Nes-Cre-ER^T2^ were found to target endothelial cells preferentially [33]. The administration of tamoxifen in neonatal Nes-Cre-ER^T2^ mice caused a higher recombination efficiency than in adults [83].

Considering this complex situation, researchers should carefully divide the Nestin^+^ cell types under microscope and select appropriate Nestin transgenic mouse models to mark MSCs.

## 9. Conclusion and Future Directions

**Figure 1:**
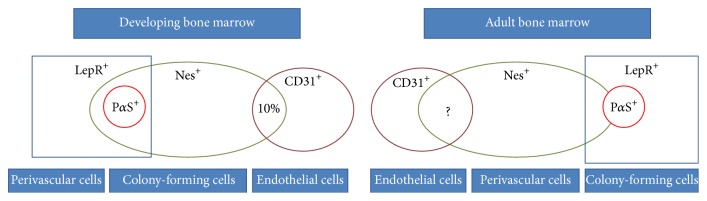
Representation of Nes^+^ cells in different developmental phases. Nes^+^ cells may represent different cell population at developmental and adult stages. Nes^+^ cells contain both endothelial and nonendothelial cells in developing bone marrow and nonendothelial Nes^+^ cells participate in endochondral ossification as MSCs. In adult bone marrow, a small portion of Nes^+^ cells overlap with LepR^+^ cells which contain most CFU-F cells while most Nes^+^ cells are vascular cells to help build blood vessel walls and maintain the niche for HSCs. The relationship between Nes^+^ cells and endothelial cells in adult is still unknown.

Although MSCs have been extensively studied according to their remarkable capacity and the enormous potential for clinical application, there is still a mass of issues remaining for characterization of these cells because of the lack of standardized protocols for isolation and unique markers for identification. Observational and interventional studies in animals and humans have shown that Nestin may be an important marker for MSCs and Nes^+^ MSCs play important roles in tissues growth and regeneration. Although Nes^+^ MSCs are quiescent in bone marrow, they preserve high colony-forming-unit fibroblastic activity and trilineage differentiation. Moreover, they are essential for HSC maintenance and mobilization via forming a structurally unique niche [47, 83, 116]. Furthermore, Nes^+^ cells represent different cell population at different developmental phases (Figure 1). They may hold the potential for angiogenesis in various tissues.

So far the knowledge on signaling pathway of regulating Nestin expression and the mechanism of mobilization and differentiation of Nes^+^ cells is limited. This leads to our unclear understanding of Nestin. Although the treatments targeting Nes^+^ cells showed an improvement in some disease animal models, more lines of evidence are needed to elucidate the real identities of Nes^+^ cells in this process. The next step should be to conduct comprehensive analyses for subdividing Nes^+^ cell population according to their specific fate and function. Researchers should carefully select the animal models to study the contribution of Nes^+^ cells* in vivo* considering that the constructs in different transgenic reporter lines of Nestin are unlike. Nevertheless, there is no doubt that Nestin is one of the best markers under steady state and with increasing attention on Nestin, we will fully characterize this protein, establish MSCs physiological and pathological function, and thus clarify the pathogenesis of diseases in the future.

## Figures and Tables

**Table 1:** Heterozygous cell population of MSC surface markers.

Surface antigen	Cell types
Stro-1	Endothelial cells [51, 52]
Sca-1 (stem cell antigen-1)	HSCs [53], cancer stem cells (CSCs) [54]
CD13 (aminopeptidase N)	CSCs [55], myeloid cells [56]
CD29 (integrin beta-1)	NSCs [57], CSCs [58]
CD44	T cells [59], CSCs [60, 61]
CD73 (NT5E)	Endothelial cells [62], lymphocytes [63]
CD90 (Thy-1)	T cells [64]
CD105 (endoglin)	HSCs [65], endothelial cells [66], macrophages [67]
CD106 (VCAM-0031)	Endothelial cells [68]
CD146 (MCAM)	T cells [69], pericytes [70], endothelial cells [71]
CD166 (ALCAM)	Epithelial cells [72, 73]
CD271 (LNGFR)	NSCs [74], CSCs [75]
PDGFR*α* (CD140a)	Fibroblasts [76], smooth muscle cells [77]
Leptin R	Adipocytes [78]

## References

[1] Cogle C. R., Guthrie S. M., Sanders R. C., Allen W. L., Scott E. W., Petersen B. E. (2003). An overview of stem cell research and regulatory issues. *Mayo Clinic Proceedings*.

[2] Clarke D. L., Johansson C. B., Wilbertz J. (2000). Generalized potential of adult neural stem cells. *Science*.

[3] Goodman J. W. (1964). Stem cells of the hematopoietic and lymphatic tissues. *National Cancer Institute Monograph*.

[4] Grottkau B. E., Purudappa P. P., Lin Y.-F. (2010). Multilineage differentiation of dental pulp stem cells from green fluorescent protein transgenic mice. *International Journal of Oral Science*.

[5] Peng L., Ye L., Zhou X.-D. (2009). Mesenchymal stem cells and tooth engineering. *International Journal of Oral Science*.

[6] Technau U., Scholz C. B. (2003). Origin and evolution of endoderm and mesoderm. *The International Journal of Developmental Biology*.

[7] Kimelman D. (2006). Mesoderm induction: from caps to chips. *Nature Reviews Genetics*.

[8] Reyes M., Lund T., Lenvik T., Aguiar D., Koodie L., Verfaillie C. M. (2001). Purification and ex vivo expansion of postnatal human marrow mesodermal progenitor cells. *Blood*.

[9] Pittenger M. F. (1999). Multilineage potential of adult human mesenchymal stem cells. *Science*.

[10] Uccelli A., Moretta L., Pistoia V. (2008). Mesenchymal stem cells in health and disease. *Nature Reviews Immunology*.

[11] Dominici M., Le Blanc K., Mueller I. (2006). Minimal criteria for defining multipotent mesenchymal stromal cells. The International Society for Cellular Therapy position statement. *Cytotherapy*.

[12] Caplan A. I. (1991). Mesenchymal stem cells. *Journal of Orthopaedic Research*.

[13] Carlson S., Trial J., Soeller C., Entman M. L. (2011). Cardiac mesenchymal stem cells contribute to scar formation after myocardial infarction. *Cardiovascular Research*.

[14] Foronjy R. F., Majka S. M. (2012). The potential for resident lung mesenchymal stem cells to promote functional tissue regeneration: understanding microenvironmental cues. *Cells*.

[15] Kim D.-W., Staples M., Shinozuka K., Pantcheva P., Kang S.-D., Borlongan C. V. (2013). Wharton's jelly-derived mesenchymal stem cells: phenotypic characterization and optimizing their therapeutic potential for clinical applications. *International Journal of Molecular Sciences*.

[16] Chong P.-P., Selvaratnam L., Abbas A. A., Kamarul T. (2012). Human peripheral blood derived mesenchymal stem cells demonstrate similar characteristics and chondrogenic differentiation potential to bone marrow derived mesenchymal stem cells. *Journal of Orthopaedic Research*.

[17] Banas A. (2012). Purification of adipose tissue mesenchymal stem cells and differentiation toward hepatic-like cells. *Methods in Molecular Biology*.

[18] Caplan A. I. (2007). Adult mesenchymal stem cells for tissue engineering versus regenerative medicine. *Journal of Cellular Physiology*.

[19] Harris D. T. (2013). Umbilical cord tissue mesenchymal stem cells: characterization and clinical applications. *Current Stem Cell Research & Therapy*.

[20] Lendahl U., Zimmerman L. B., McKay R. D. G. (1990). CNS stem cells express a new class of intermediate filament protein. *Cell*.

[21] Dahlstrand J., Lardelli M., Lendahl U. (1995). Nestin mRNA expression correlates with the central nervous system progenitor cell state in many, but not all, regions of developing central nervous system. *Developmental Brain Research*.

[22] Di C. G., Xiang A. P., Jia L., Liu J. F., Lahn B. T., Ma B. F. (2014). Involvement of extracellular factors in maintaining self-renewal of neural stem cell by nestin. *NeuroReport*.

[23] Suzuki S., Namiki J., Shibata S., Mastuzaki Y., Okano H. (2010). The neural stem/progenitor cell marker nestin is expressed in proliferative endothelial cells, but not in mature vasculature. *The Journal of Histochemistry and Cytochemistry*.

[24] Krupkova O., Loja T., Zambo I., Veselska R. (2010). Nestin expression in human tumors and tumor cell lines. *Neoplasma*.

[25] Kishaba Y., Matsubara D., Niki T. (2010). Heterogeneous expression of nestin in myofibroblasts of various human tissues. *Pathology International*.

[26] Abe S., Hamada K., Miura M., Yamaguchi S. (2012). Neural crest stem cell property of apical pulp cells derived from human developing tooth. *Cell Biology International*.

[27] Du K., Peng Y., Zhang L., Liang A., Huang D. (2011). Expression of the stem cell marker nestin in pre/hypertrophic chondrocytes in osteochondroma. *Journal of International Medical Research*.

[28] Wiese C., Rolletschek A., Kania G. (2004). Nestin expression—a property of multi-lineage progenitor cells?. *Cellular and Molecular Life Sciences*.

[29] Zulewski H., Abraham E. J., Gerlach M. J. (2001). Multipotential nestin-positive stem cells isolated from adult pancreatic islets differentiate ex vivo into pancreatic endocrine, exocrine, and hepatic phenotypes. *Diabetes*.

[30] Kunisaki Y., Bruns I., Scheiermann C. (2013). Arteriolar niches maintain haematopoietic stem cell quiescence. *Nature*.

[31] Méndez-Ferrer S., Michurina T. V., Ferraro F. (2010). Mesenchymal and haematopoietic stem cells form a unique bone marrow niche. *Nature*.

[32] Mendelson A., Frenette P. S. (2014). Hematopoietic stem cell niche maintenance during homeostasis and regeneration. *Nature Medicine*.

[33] Ono N., Ono W., Mizoguchi T., Nagasawa T., Frenette P. S., Kronenberg H. M. (2014). Vasculature-associated cells expressing nestin in developing bones encompass early cells in the osteoblast and endothelial lineage. *Developmental Cell*.

[34] Matsuda Y., Hagio M., Ishiwata T. (2013). Nestin: a novel angiogenesis marker and possible target for tumor angiogenesis. *World Journal of Gastroenterology*.

[35] Steinert P. M., Liem R. K. H. (1990). Intermediate filament dynamics. *Cell*.

[36] Michalczyk K., Ziman M. (2005). Nestin structure and predicted function in cellular cytoskeletal organisation. *Histology and Histopathology*.

[37] Karabinos A., Riemer D., Erber A., Weber K. (1998). Homologues of vertebrate type I, II and III intermediate filament (IF) proteins in an invertebrate: the IF multigene family of the cephalochordate *Branchiostoma*. *FEBS Letters*.

[38] Herrmann H., Bär H., Kreplak L., Strelkov S. V., Aebi U. (2007). Intermediate filaments: from cell architecture to nanomechanics. *Nature Reviews Molecular Cell Biology*.

[39] Guérette D., Khan P. A., Savard P. E., Vincent M. (2007). Molecular evolution of type VI intermediate filament proteins. *BMC Evolutionary Biology*.

[40] Buchkovich N. J., Henne W. M., Tang S., Emr S. D. (2013). Essential N-Terminal insertion motif anchors the ESCRT-III filament during MVB vesicle formation. *Developmental Cell*.

[41] Steinert P. M., Chou Y.-H., Prahlad V. (1999). A high molecular weight intermediate filament-associated protein in BHK-21 cells is nestin, a type VI intermediate filament protein. Limited co-assembly in vitro to form heteropolymers with type III vimentin and type IV alpha-internexin. *The Journal of Biological Chemistry*.

[42] Marvin M. J., Dahlstrand J., Lendahl U., McKay R. D. G. (1998). A rod end deletion in the intermediate filament protein nestin alters its subcellular localization in neuroepithelial cells of transgenic mice. *Journal of Cell Science*.

[43] Sahlgren C. M., Mikhailov A., Hellman J. (2001). Mitotic reorganization of the intermediate filament protein nestin involves phosphorylation by cdc2 kinase. *Journal of Biological Chemistry*.

[44] Schipani E., Kronenberg H. M. (2008). Adult mesenchymal stem cells. *StemBook*.

[45] Boxall S. A., Jones E. (2012). Markers for characterization of bone marrow multipotential stromal cells. *Stem Cells International*.

[46] Tondreau T., Lagneaux L., Dejenefle M. (2004). Bone marrow-derived mesenchymal stem cells already express specific neural proteins before any differentiation. *Differentiation*.

[47] Isern J., Méndez-Ferrer S. (2011). Stem cell interactions in a bone marrow niche. *Current Osteoporosis Reports*.

[48] Pillozzi S., Becchetti A. (2012). Ion channels in hematopoietic and mesenchymal stem cells. *Stem Cells International*.

[49] Pinho S., Lacombe J., Hanoun M. (2013). PDGFR*α* and CD51 mark human Nestin^+^ sphere-forming mesenchymal stem cells capable of hematopoietic progenitor cell expansion. *The Journal of Experimental Medicine*.

[50] Crisan M., Yap S., Casteilla L. (2008). A perivascular origin for mesenchymal stem cells in multiple human organs. *Cell Stem Cell*.

[51] Lin G., Liu G., Banie L. (2011). Tissue distribution of mesenchymal stem cell marker stro-1. *Stem Cells and Development*.

[52] Ning H., Lin G., Lue T. F., Lin C.-S. (2011). Mesenchymal stem cell marker Stro-1 is a 75 kd endothelial antigen. *Biochemical and Biophysical Research Communications*.

[53] Bradfute S. B., Graubert T. A., Goodell M. A. (2005). Roles of Sca-1 in hematopoietic stem/progenitor cell function. *Experimental Hematology*.

[54] Grange C., Lanzardo S., Cavallo F., Camussi G., Bussolati B. (2008). SCA-1 identifies the tumor-initiating cells in mammary tumors of BALB-neuT trangenic mice. *Neoplasia*.

[55] Haraguchi N., Ishii H., Mimori K. (2010). CD13 is a therapeutic target in human liver cancer stem cells. *The Journal of Clinical Investigation*.

[56] Dondossola E., Rangel R., Guzman-Rojas L. (2013). CD13-positive bone marrow-derived myeloid cells promote angiogenesis, tumor growth, and metastasis. *Proceedings of the National Academy of Sciences of the United States of America*.

[57] Pruszak J., Ludwig W., Blak A., Alavian K., Isacson O. (2009). CD15, CD24, and CD29 define a surface biomarker code for neural lineage differentiation of stem cells. *Stem Cells*.

[58] Geng S., Guo Y., Wang Q., Li L., Wang J. (2013). Cancer stem-like cells enriched with CD29 and CD44 markers exhibit molecular characteristics with epithelial-mesenchymal transition in squamous cell carcinoma. *Archives of Dermatological Research*.

[59] Baaten B. J. G., Li C.-R., Bradley L. M. (2010). Multifaceted regulation of T cells by CD44. *Communicative & Integrative Biology*.

[60] Hiraga T., Ito S., Nakamura H. (2013). Cancer stem-like cell marker CD44 promotes bone metastases by enhancing tumorigenicity, cell motility, and hyaluronan production. *Cancer Research*.

[61] De Beca F. F., Caetano P., Gerhard R. (2013). Cancer stem cells markers CD44, CD24 and ALDH1 in breast cancer special histological types. *Journal of Clinical Pathology*.

[62] Narravula S., Lennon P. F., Mueller B. U., Colgan S. P. (2000). Regulation of endothelial CD73 by adenosine: paracrine pathway for enhanced endothelial barrier function. *The Journal of Immunology*.

[63] Mills J. H., Thompson L. F., Mueller C. (2008). CD73 is required for efficient entry of lymphocytes into the central nervous system during experimental autoimmune encephalomyelitis. *Proceedings of the National Academy of Sciences of the United States of America*.

[64] Rege T. A., Hagood J. S. (2006). Thy-1 as a regulator of cell-cell and cell-matrix interactions in axon regeneration, apoptosis, adhesion, migration, cancer, and fibrosis. *The FASEB Journal*.

[65] Pierelli L., Bonanno G., Rutella S., Marone M., Scambia G., Leone G. (2001). CD105 (endoglin) expression on hematopoietic stem/progenitor cells. *Leukemia & Lymphoma*.

[66] Yoshitomi H., Kobayashi S., Ohtsuka M. (2008). Specific expression of endoglin (CD105) in endothelial cells of intratumoral blood and lymphatic vessels in pancreatic cancer. *Pancreas*.

[67] Ren S., Fan X., Peng L. (2013). Expression of NF-*κ*B, CD68 and CD105 in carotid atherosclerotic plaque. *Journal of Thoracic Disease*.

[68] Heidemann J., Maaser C., Lügering A. (2006). Expression of vascular cell adhesion molecule-1 (CD 106) in normal and neoplastic human esophageal squamous epithelium. *International Journal of Oncology*.

[69] Hadjinicolaou A. V., Wu L., Fang B., Watson P. A., Hall F. C., Busch R. (2013). Relationship of CD146 expression to activation of circulating T cells: exploratory studies in healthy donors and patients with connective tissue diseases. *Clinical and Experimental Immunology*.

[70] Murray I. R., West C. C., Hardy W. R. (2014). Natural history of mesenchymal stem cells, from vessel walls to culture vessels. *Cellular and Molecular Life Sciences*.

[71] Li Q., Yu Y., Bischoff J., Mulliken J. B., Olsen B. R. (2003). Differential expression of CD146 in tissues and endothelial cells derived from infantile haemangioma and normal human skin. *The Journal of Pathology*.

[72] Fujiwara H., Tatsumi K., Kosaka K. (2003). Human blastocysts and endometrial epithelial cells express activated leukocyte cell adhesion molecule (ALCAM/CD166). *The Journal of Clinical Endocrinology and Metabolism*.

[73] Abidi S. M. A., Saifullah M. K., Zafiropulos M. D. (2006). CD166 expression, characterization, and localization in salivary epithelium: implications for function during sialoadenitis. *Journal of Clinical Immunology*.

[74] Dupin E., Coelho-Aguiar J. M. (2013). Isolation and differentiation properties of neural crest stem cells. *Cytometry Part A*.

[75] Boiko A. D., Razorenova O. V., van de Rijn M. (2010). Human melanoma-initiating cells express neural crest nerve growth factor receptor CD271. *Nature*.

[76] Erez N., Truitt M., Olson P., Hanahan D. (2010). Cancer-associated fibroblasts are activated in incipient neoplasia to orchestrate tumor-promoting inflammation in an NF-*κ*B-dependent manner. *Cancer Cell*.

[77] Hu Y., Böck G., Wick G., Xu Q. (1998). Activation of PDGF receptor alpha in vascular smooth muscle cells by mechanical stress. *The FASEB Journal*.

[78] Kielar D., Clark J. S. C., Ciechanowicz A., Kurzawski G., Sulikowski T., Naruszewicz M. (1998). Leptin receptor isoforms expressed in human adipose tissue. *Metabolism: Clinical and Experimental*.

[79] Lucas D., Bruns I., Battista M. (2012). Norepinephrine reuptake inhibition promotes mobilization in mice: potential impact to rescue low stem cell yields. *Blood*.

[80] Ding L., Saunders T. L., Enikolopov G., Morrison S. J. (2012). Endothelial and perivascular cells maintain haematopoietic stem cells. *Nature*.

[81] Tronche F., Kellendonk C., Kretz O. (1999). Disruption of the glucocorticoid receptor gene in the nervous system results in reduced anxiety. *Nature Genetics*.

[82] Mizoguchi T., Pinho S., Ahmed J. (2014). Osterix marks distinct waves of primitive and definitive stromal progenitors during bone marrow development. *Developmental Cell*.

[83] Isern J., García-García A., Martín A. M. (2014). The neural crest is a source of mesenchymal stem cells with specialized hematopoietic stem cell niche function. *eLife*.

[84] Oswald J., Boxberger S., Jørgensen B. (2004). Mesenchymal stem cells can be differentiated into endothelial cells in vitro. *Stem Cells*.

[85] Jazayeri M., Allameh A., Soleimani M., Jazayeri S. H., Piryaei A., Kazemnejad S. (2008). Molecular and ultrastructural characterization of endothelial cells differentiated from human bone marrow mesenchymal stem cells. *Cell Biology International*.

[86] Liu J. W., Dunoyer-Geindre S., Serre-Beinier V. (2007). Characterization of endothelial-like cells derived from human mesenchymal stem cells. *Journal of Thrombosis and Haemostasis*.

[87] Ball S. G., Worthington J. J., Canfield A. E., Merry C. L. R., Kielty C. M. (2014). Mesenchymal stromal cells: inhibiting PDGF receptors or depleting fibronectin induces mesodermal progenitors with endothelial potential. *Stem Cells*.

[88] Henrich D., Wilhelm K., Warzecha J. (2013). Human endothelial-like differentiated precursor cells maintain their endothelial characteristics when cocultured with mesenchymal stem cell and seeded onto human cancellous bone. *Mediators of Inflammation*.

[89] Klein D., Meissner N., Kleff V., Jastrow H., Yamaguchi M., Jendrossek V. (2014). Nestin(+) tissue-resident multipotent stem cells contribute to tumor progression by differentiating into pericytes and smooth muscle cells resulting in blood vessel remodeling. *Frontiers in Oncology*.

[90] Mokrý J., Němeček S. (1999). Cerebral angiogenesis shows nestin expression in endothelial cells. *General Physiology and Biophysics*.

[91] Yamahatsu K., Matsuda Y., Ishiwata T., Uchida E., Naito Z. (2012). Nestin as a novel therapeutic target for pancreatic cancer via tumor angiogenesis. *International Journal of Oncology*.

[92] El-Helou V., Beguin P. C., Assimakopoulos J. (2008). The rat heart contains a neural stem cell population; Role in sympathetic sprouting and angiogenesis. *Journal of Molecular and Cellular Cardiology*.

[93] Sugawara K.-I., Kurihara H., Negishi M. (2002). Nestin as a marker for proliferative endothelium in gliomas. *Laboratory Investigation*.

[94] You H., Kim Y. I., Im S. Y. (2005). Immunohistochemical study of central neurocytoma, subependymoma, and subependymal giant cell astrocytoma. *Journal of Neuro-Oncology*.

[95] Veselska R., Hermanova M., Loja T. (2008). Nestin expression in osteosarcomas and derivation of nestin/CD133 positive osteosarcoma cell lines. *BMC Cancer*.

[96] Ishiwata T., Matsuda Y., Naito Z. (2011). Nestin in gastrointestinal and other cancers: effects on cells and tumor angiogenesis. *World Journal of Gastroenterology*.

[97] Teranishi N., Naito Z., Ishiwata T. (2007). Identification of neovasculature using nestin in colorectal cancer. *International Journal of Oncology*.

[98] Bianco P., Cao X., Frenette P. S. (2013). The meaning, the sense and the significance: translating the science of mesenchymal stem cells into medicine. *Nature Medicine*.

[99] Au P., Tam J., Fukumura D., Jain R. K. (2008). Bone marrow-derived mesenchymal stem cells facilitate engineering of long-lasting functional vasculature. *Blood*.

[100] Petrini M., Pacini S., Trombi L. (2009). Identification and purification of mesodermal progenitor cells from human adult bone marrow. *Stem Cells and Development*.

[101] Pacini S., Carnicelli V., Trombi L. (2010). Constitutive expression of pluripotency-associated genes in mesodermal progenitor cells (MPCs). *PLoS ONE*.

[102] Pacini S., Petrini I. (2014). Are MSCs angiogenic cells? New insights on human nestin-positive bone marrow-derived multipotent cells. *Frontiers in Cell and Developmental Biology*.

[103] Crane J. L., Cao X. (2014). Bone marrow mesenchymal stem cells and TGF-*β* signaling in bone remodeling. *The Journal of Clinical Investigation*.

[104] Zhen G., Wen C., Jia X. (2013). Inhibition of TGF-*β* signaling in mesenchymal stem cells of subchondral bone attenuates osteoarthritis. *Nature Medicine*.

[105] Hanoun M., Zhang D., Mizoguchi T. (2014). Acute myelogenous leukemia-induced sympathetic neuropathy promotes malignancy in an altered hematopoietic stem cell niche. *Cell Stem Cell*.

[106] Arranz L., Sánchez-Aguilera A., Martín-Pérez D. (2014). Neuropathy of haematopoietic stem cell niche is essential for myeloproliferative neoplasms. *Nature*.

[107] Baryawno N., Scadden D. T. (2014). Blood loses it when nerves go bad. *Cell Research*.

[108] Gao P., Zhou Y., Xian L. (2014). Functional effects of TGF-*β*1 on mesenchymal stem cell mobilization in cockroach allergen-induced asthma. *Journal of Immunology*.

[109] Wan M., Li C., Zhen G. (2012). Injury-activated transforming growth factor *β* controls mobilization of mesenchymal stem cells for tissue remodeling. *Stem Cells*.

[110] Wong A., Ghassemi E., Yellowley C. E. (2014). Nestin expression in mesenchymal stromal cells: Regulation by hypoxia and osteogenesis. *BMC Veterinary Research*.

[111] Wislet-Gendebien S., Laudet E., Neirinckx V. (2012). Mesenchymal stem cells and neural crest stem cells from adult bone marrow: characterization of their surprising similarities and differences. *Cellular and Molecular Life Sciences*.

[112] Zhou B. O., Yue R., Murphy M. M., Peyer J. G., Morrison S. J. (2014). Leptin-receptor-expressing mesenchymal stromal cells represent the main source of bone formed by adult bone marrow. *Cell Stem Cell*.

[113] Worthley D. L., Churchill M., Compton J. T. (2015). Gremlin 1 identifies a skeletal stem cell with bone, cartilage, and reticular stromal potential. *Cell*.

[114] Mignone J. L., Kukekov V., Chiang A.-S., Steindler D., Enikolopov G. (2004). Neural stem and progenitor cells in nestin-GFP transgenic mice. *The Journal of Comparative Neurology*.

[115] Balordi F., Fishell G. (2007). Mosaic removal of hedgehog signaling in the adult SVZ reveals that the residual wild-type stem cells have a limited capacity for self-renewal. *The Journal of Neuroscience*.

[116] Frenette P. S., Pinho S., Lucas D., Scheiermann C. (2013). Mesenchymal stem cell: keystone of the hematopoietic stem cell niche and a stepping-stone for regenerative medicine. *Annual Review of Immunology*.

